# Stoma-Const - the technical aspects of stoma construction: study protocol for a randomised controlled trial

**DOI:** 10.1186/1745-6215-15-254

**Published:** 2014-06-27

**Authors:** Adiela Correa Marinez, Sofia Erestam, Eva Haglind, Jan Ekelund, Ulf Angerås, Jacob Rosenberg, Frederik Helgstrand, Eva Angenete

**Affiliations:** 1 Department of Surgery, Institute of Clinical Sciences, SSORG - Scandinavian Surgical Outcomes Research Group, University of Gothenburg, Sahlgrenska University Hospital/Östra, 416 85 Gothenburg, Sweden; 2 Department of Anaesthesia, Surgery and Intensive Care, Sahlgrenska University Hospital/Östra, SE-416 85 Gothenburg, Sweden; 3 Department of Surgery, Herlev University Hospital, Copenhagen, Denmark; 4 Department of Surgical Gastroenterology, Rigshospitalet, Copenhagen, Denmark

**Keywords:** Parastomal hernia, Colostomy, Mesh, Prevention

## Abstract

**Background:**

The construction of a colostomy is a common procedure, but the evidence for the different parts of the construction of the colostomy is lacking. Parastomal hernia is a common complication of colostomy formation. The aim of this study is to standardise the colostomy formation and to compare three types of colostomy formation (one including a mesh) regarding the development of parastomal hernia.

**Methods/Design:**

Stoma-Const is a Scandinavian randomised trial comparing three types of colostomy formation. The primary endpoint is parastomal herniation as shown by clinical examination or CT scan within one year. Secondary endpoints are re-admission rate, postoperative complications (classified according to Clavien-Dindo), stoma-related complications (registered in the case record form at stoma care nurse follow-up), total length of hospital stay during 12 months, health-related quality of life and health economic analysis as well as re-operation rate and mortality within 30 days and 12 months of primary surgery. Follow-up is scheduled at 4-6 weeks, and 6 and 12 months. Inclusion is set at 240 patients.

**Discussion:**

Parastomal hernia is a common complication after colostomy formation. Several studies have been performed with the aim to reduce the rate of this complication. However, none are fully conclusive and data on quality of life and health economy are lacking. The aim of this study is to develop new standardised techniques for colostomy formation and evaluate this with patient reported outcomes as well as clinical and radiological assessment.

**Trial registration:**

Clinicaltrials.gov, NCT01694238.2012-09-24.

## Background

Colostomy formation has been a standard surgical procedure for more than 100 years. A well-functioning colostomy may not negatively affect the patient’s quality of life (QoL) [1]; however, this can only be said if the stoma is well functioning and if complications are kept to a minimum. The complication rates after stoma formation are considerable, with figures of 21-70% [2,3], and studies have shown that adequate stoma height, type of stoma, body mass index (BMI), emergency surgery and gender may be of importance in reducing the risk of complications in both the short- and long-term perspectives [4-6].

The surgical technique of stoma formation is only partly evidence based. There are few studies focused on technical details about stoma construction and their future impact on stoma function. One study has attempted to standardise the skin incision to two-thirds of the width of the bowel [7], although the actual impact of this on the functional outcome of the stoma function was not presented. In the surgical literature a cruciate incision in the fascia and extraction of the bowel through a hole sufficient in size is a standard description of the surgical technique [8]. In clinical practice the sufficient size of the hole has often been equal to ‘two fingers-width’ , which is a fairly inexact measurement.

Parastomal hernia is a long-term complication that is common; in the literature figures up to almost 50% have been reported [9,10]. There have been discussions regarding the placement of the stoma and effects on hernia incidence [11,12]; however, no studies have been sufficient in design or size to thoroughly answer the question. Attempts to reduce the rates of parastomal hernias have been made in the last few years with the placement of a mesh at the construction of the stoma [13-18]. However, these trials did not include studies of health-related QoL or health economic aspects. The placement of a prophylactic parastomal mesh has not been universally accepted, mainly due to a hesitance related to the possible risk of infection with a foreign body, but also as most studies were small in sample size [19]. Another suggestion for the basic construction of the stoma has been to make a circular instead of a cruciate incision in the fascia, but this has not been studied. It has been described in conjunction with the use of circular stapling devices, and no hernias were found, but the studies were small [20,21]. Thus, it is apparent that further studies are desired [17,22-24] and several randomised controlled trials are registered and ongoing.

The evaluation of parastomal hernias can be performed by clinical examination, CT scan or ultrasound [14,15,25,26]. All techniques have advantages and disadvantages, and the conclusion must be that evaluation of parastomal hernias may be difficult and must be standardised in a study.

The hypothesis to be tested in this study is that it is possible to obtain a lower incidence of parastomal hernia with a circular incision in the abdominal fascia or with reinforcement with a mesh instead of a cruciate incision in the abdominal fascia.

The study will provide a precise control compared to the two interventions with thorough measurements of the bowel and the trephine opening to improve the evaluation of the surgical technique. It will also enable an evaluation of QoL and health economy.

## Methods

### Study objective

The Stoma-Const trial is a randomised trial comparing three different surgical procedures for the construction of a colostomy.

### Endpoints

The primary endpoint is the presence of parastomal hernia as detected by clinical examination or CT scan at 12 months after surgery. A bulge in the vicinity of the stoma is not sufficient in this study to define a parastomal hernia. If there is any doubt in the clinical setting, the patients must be examined by a CT scan in a prone position. Secondary endpoints include: re-admission rate, postoperative complications (classified according to Clavien-Dindo [27]), stoma-related complications (registered in the case record form (CRF) at Stoma Care nurse follow-up), total length of hospital stay during 12 months, health-related quality of life and health economic analysis, re-operation rate, mortality within 30 days and 12 months of primary surgery. Re-admissions and re-operations as registered in the hospitals’ databases at 24 months to ascertain that possible long-term complications related to the stoma construction are identified.

### Inclusion criteria

Patients are eligible if the following conditions are met:

•the patient is presenting with a condition for which an elective surgical procedure is planned including formation of a colostomy

•it is possible to operate on the patient in regard to concomitant disease

•the patient has given informed consent to participate

### Exclusion criteria

•Participation in other randomised trials in conflict with the protocol and endpoints of the Stoma-Const trial.

### External validity

All patients with a condition for which an elective surgical procedure is planned including a colostomy formation, and who are not included and randomised, will be registered in the ‘screening log’ at each participating centre including information on date, hospital, gender, age, ASA classification and type of operation. The reason for non-inclusion or exclusion is noted.

### Randomisation

**Figure 1:**
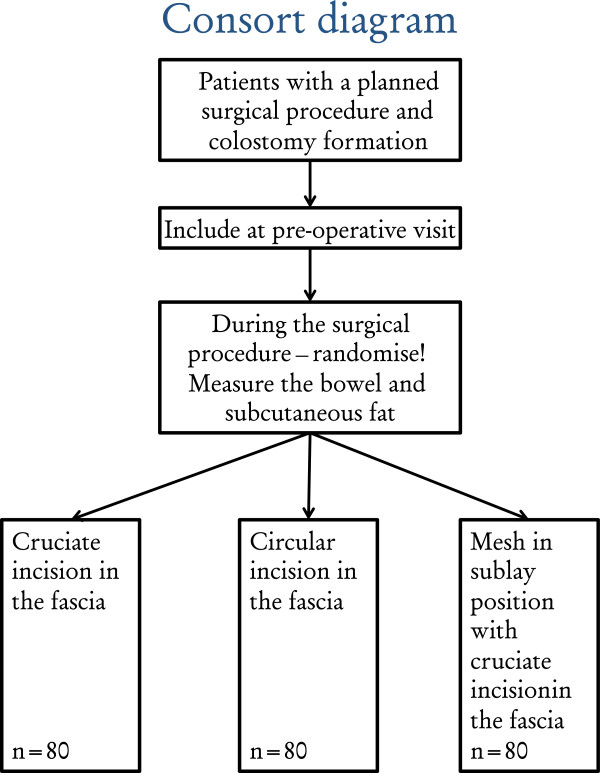
Consort diagram for the Stoma-Const trial.

All participating patients give written informed consent. After inclusion no changes are made in the planning for the operation. The patient will be randomised to one of the three groups of intervention during surgery in the operating theatre directly prior to the stoma formation (Figure 1). Randomisation will be performed by blocks of closed envelope systems in each participating hospital. It will be balanced and stratified by hospital. Laparoscopic as well as open procedures will be included and, although not stratified by open or laparoscopic procedures, their use will be noted in the CRF.

Including hospitals may choose to randomise between all three techniques or two of them. This decision is made before the hospital enters the study. The blocks will be set up according to this system, and the total number of 80 patients in each group will be managed through this system.

When patients are not subject to the treatment modality as randomised, data will be analysed on an ‘intention-to-treat basis’ (once randomised, patients will not be excluded or change groups because of conversion or type of surgery). Patients who do not consent to participation or are excluded will be treated by cross incision in the fascia as the usual routine.

### Surgical procedure

A stoma care nurse marks all stoma sites preoperatively. At the time of the stoma formation, randomisation to one of the three (or in applicable cases two) procedures is made. The centre point of the preoperatively marked place for the stoma is lifted and a circular skin incision is performed. Blunt or ‘semi-blunt’ dissection is used through the subcutaneous tissue, using a step-wise downwards movement with retractors assisted by sharp dissection as needed. When the ventral fascia of the rectus abdominis has been reached, dissection is stopped. A disposable, sterile measurement tape is used in the open surgical procedures, and the metric scale on the laparoscopic stapler is used in laparoscopic surgical procedures. The depth of the subcutaneous fat is measured. The width of the left colon together with its mesocolon is measured at the point where it will pass through the fascia (the depth of the subcutaneous fat + 20-30 mm). The fascia incision is calculated using this measurement - circular incision in the fascia with a diameter equalling 50% of the width of the patient’s left colon with mesocolon, or a cruciate incision where the arms measure one-half of the width of the patient’s left colon with mesocolon. If it is apparent that the incision will be too large, it may be reduced, but measured thoroughly and noted in the operative CRF. The muscular fibres are separated bluntly.

#### Stoma formation without a mesh

The dorsal fascia is opened. The proximal colon end is taken through the stoma incision (plica epiploicae may be removed, if necessary). Care is taken to ensure adequate circulation to the colon involved in the stoma. If the width of the incision is insufficient for adequate circulation, a widening should be performed, measured and noted in the CRF. The muco-cutaneous suturing is made using 4-0 absorbable interrupted sutures, three-point sutures (mucosa, muscular layer and skin) starting with one in each quadrant and adding ordinary muco-cutaneous sutures in between as needed. The height of the stoma after it is created should be at least 1 cm.

#### Stoma formation with a mesh

The incision in the fascia is performed with a cruciate incision, with size according to the measurements described above, and the mesh will be placed using the sublay technique as previously described [14]. A fine-thread, large-pore lightweight polypropylene monofilament mesh measuring 10 × 10 cm is used. The mesh is placed dorsal to the rectus abdominis muscle and anterior to the posterior rectus fascia by dissection through either midline in open surgery or by blunt dissection under the rectus abdominis muscle in laparoscopic surgery. A cruciate incision with a size measured according to the instructions above is made in the anterior sheath of the fascia and in the mesh. The bowel is brought out through a cross-cut in the centre of the mesh where the arms should measure one-half of the width of the patient’s left colon and its mesocolon. If the width of the incision is not sufficient, a widening should be performed, measured and noted in the CRF. Stitches fix the lateral corners of the mesh to the posterior rectus sheath. The medial corners of the mesh are grasped with a stitch of the running suture closing the midline incision during open surgery, and this is not applicable to laparoscopic procedures.

The diameter of the stoma is measured immediately after the operation.

### Postoperative treatment

Local guidelines are followed and must be applied equally in all three (in applicable cases, two) groups, without any systematic differences. Any postoperative treatment with antibiotics must be noted in the CRF, including reasons and total treatment time. This applies to both systemic as well as oral antibiotics. All postoperative information regarding stoma and stoma function will be given according to local routines.

### Follow-up

The follow-up during the postoperative hospital stay is recorded in a CRF. Time until full oral feeding is resumed and time until first gas and stool are registered.

Patients will be clinically examined at 6 (5–7) months and at 12 (11–13) months postoperatively and further as needed (suspected parastomal herniation), by a surgeon (not one of the colorectal team) preferably specialised in hernia surgery, to diagnose a possible parastomal hernia. At 12 months patients for whom no clinical parastomal herniation has been found will undergo a CT examination of the abdomen (wall), performed in prone position [25], which also will be part of the routine 12 months follow-up by abdomen CT for colorectal cancer patients. A study-specific standardised referral to abdomen CT is used, and although the study is not blinded, no information as to type of stoma construction is included. All radiologic departments use a specific protocol for this part of the abdomen CT.

Stoma height, diameter, colour, skin irritation and bandaging problems are documented by nurses specialised in stoma care during hospitalisation and at 4-6 weeks, 6 months and 12 months and are registered in a CRF.

### Quality of life

All patients are asked to fill out questionnaires preoperatively, at 6 months and at 12 months after discharge from the hospital. The questionnaire has been constructed using previously used questions [28] that have been developed through a process of in-depth qualitative interviews and content validated by an expert panel consisting of colorectal surgeons, stoma care nurses and nurses specialised in surgery. The questionnaire was face-to-face validated by patients with a surgical procedure including a colostomy as well as patients with an existing colostomy using the validation methods described previously for a questionnaire for prostate cancer patients (26).

### Health economic evaluation

A health economic analysis will be performed based on the information collected in the CRF together with information from the hospital registries, based on the model presented by Bjöholt *et al.*[29]. The model will be used in combination with sensitivity calculations.

### Data collecting and monitoring

An operative CRF including reasons for surgery, planned operation and patient data, together with measurements from the surgical procedure is filled in for the operation (by the surgeon). For the hospitalisation period a nurse at the ward will complete a CRF, and for each follow-up visit a surgeon and a nurse will complete a CRF.

The postoperative questionnaires will be given to the patients by the stoma therapist. All data will be kept within University of Gothenburg and Sahlgrenska University Hospital systems using inherent security systems. A logistic database with complete patient ID will be used and kept within a separate IT system from the result database with all study information. Security measures will include one to maximum two users of this database, with unique usernames and personal login, as well as automatic throw-out. The questionnaires filled out by the patients are returned to the trial coordinating centre, SSORG, at Sahlgrenska University Hospital. The finalised database based on trial number and without patient ID, will be kept within another IT system than the logistic database.

### Statistical analysis

The Swedish trial on the effect of prophylactic net placement has reported 50% incidence of parastomal hernia without mesh [14]. Our own data on abdominoperineal excision from Sahlgrenska University Hospital indicate an incidence of 25% [28]. The power calculation based on available information has used the hypothesis that the cruciate incisional stoma formation, performed as described here, should result in parastomal hernia incidence of 30% and the circular incision or prophylactic mesh reinforcement could reduce this incidence to 10% each. An 80% power and a 5% level of significance with a two-sided alternative hypothesis would require 62 patients per group using a chi-square test. Expecting 20% drop-outs, 80 patients will be recruited in each group.

The primary analysis will be based on the full analysis set. This analysis set is designed to be consistent with recommendations set forth in International Conference on Harmonisation (ICH) guidance document E9: Statistical Principles for Clinical Trials, and is intended to adhere as closely as practically possible to the intention-to-treat (ITT) principle. The full analysis set will consist of all randomised patients who had surgery and who contributed with any follow-up data.

Patients will be analysed according to randomised procedure. A sensitivity analysis evaluating the actual procedure will be made for the most important outcomes.

All hypothesis testing will be conducted using two-sided tests, and P values ≤ 0.05 after rounding will be considered statistically significant. A pair-wise comparison between the experimental arms and the control arm will be performed and the significance level will be corrected using the Bonferroni-Holm method. Event-type variables (yes or no) will be compared between randomised procedures using chi-square tests, and relative risks and risk differences will be estimated. Where appropriate, the time to event will be described using Kaplan-Meier plots. The number of events of a certain type (for example, number of complications or re-operations) will be analysed using Poisson regression with randomised procedure as a factor and time under observation as an offset variable.

Total length of hospital stay will be compared between groups using Wilcoxon’s rank-sum test. Sub-group analyses specifically looking at the following groups of patients will be performed:

a) BMI >30 kg/m2

b) Immunosuppression

Sensitivity analyses will be performed for the primary variable and selected secondary variables, as deemed appropriate, where groups are compared pair-wise utilising only data from centres where both treatments were allocated.

Exploratory analyses (such as logistic regression) may be performed to investigate the influence of background factors (patient characteristics, perioperative factors) on outcomes.

### Participating hospitals

The study will recruit patients from hospitals in Denmark and Sweden.

### Approvals and registration

The trial has been approved by the Swedish Ethical Committee (EPN/Göteborg Dnr 547-12) and the Swedish radiotherapy protection committee (Dnr 12-38). It has been approved by the Danish ethics committee (Protocol H-4-2013-061), and by the Danish Data Protection Agency (no. HEH-2013-049, I-Suite no:02418). The study was registered at http://www.clinicaltrials.gov (NCT NCT01694238) prior to inclusion of the first patient.

## Discussion

Parastomal hernia is a common complication after stoma creation (12, 13). There are several randomised trials that have tried to evaluate a reduction of the occurence of parastomal hernia with placement of a prophylactic mesh [13,14,30]. However, the use of a prophylactic measure must be evaluated in relation to health-related QoL as well as health economy. It is important to have enough power to thoroughly evaluate possible complications and side effects. A recent health technology assessment at Sahlgrenska University Hospital concluded that mesh probably is beneficial in regard to parastomal hernia formation, but that further studies are required [31]. At present there are at least six other randomised trials investigating the importance of a mesh for prevention of parastomal hernia, including one Norweigan study [32] with 60 patients, one Danish study that was stopped before including the planned 198 patients [33] and one Swedish study with 300 patients [34]. Sixty patients are randomised to mesh/no mesh in a Spanish study [35], another Spanish study is randomising 32 patients to intraperitoneal mesh placement [36] and 200 patients are randomised in a French study [37]. In addition there is a Dutch study, with 150 patients [38].

Our study will provide additional information complementing these studies through the evaluation of the surgical technique without a mesh in the control arm and in the interventional arms. Furthermore, the QoL and health economic evaluation will enable improved decision-making regarding prophylactic measures against parastomal herniation.

## Trial status

The trial is ongoing and recruiting patients at centres in both Denmark and Sweden. Hospitals interested in participating are welcome to contact the corresponding author.

## Abbreviations

ASA classification: American Society of Anaesthesiology classification; BMI: body mass index; CRF: case record form; CT: computed tomography; EPN: Ethical committee; ICH: International Conference on Harmonisation; ID: identification; IT: information technology; ITT: intention to treat; QoL: quality of life.

## Competing interests

The authors declare that they have no competing interests.

## Authors’ contributions

ACM participated in the design of the study and the study protocol, CRFs and questionnaires, and the statistical considerations as well as in drafting of the manuscript. SE participated in the preparation of the study protocol, CRFs and questionnaires and drafting of the manuscript. EH conceived the study, participated in the design of the study and the study protocol, CRFs and questionnaires and the statistical considerations as well as in drafting of the manuscript. JE performed the statistical analysis plan and the drafting of the manuscript. UA participated in the design of the study and the study protocol as well as drafting of the manuscript. JR participated in the design of the study and the study protocol as well as drafting of the manuscript. FH participated in the design of the study and the study protocol as well as drafting of the manuscript. EA participated in the design of the study and the study protocol, CRFs and questionnaires and the statistical considerations and performed the drafting of the manuscript. All authors read and approved the final manuscript.

## References

[B1] Pachler J Wille-Jorgensen P Quality of life after rectal resection for cancer, with or without permanent colostomy Cochrane Database Syst Rev 2012 12 CD004323 10.1002/14651858.CD004323.pub4 PMC7197443 23235607

[B2] Shabbir J Britton DC Stoma complications: a literature overview Colorectal Dis 2010 12 958 964 19604288 10.1111/j.1463-1318.2009.02006.x

[B3] Porter JA Salvati EP Rubin RJ Eisenstat TE Complications of colostomies Dis Colon Rectum 1989 32 299 303 2924670 10.1007/BF02553484

[B4] Cottam J Richards K Hasted A Blackman A Results of a nationwide prospective audit of stoma complications within 3 weeks of surgery Colorectal Dis 2007 9 834 838 17672873 10.1111/j.1463-1318.2007.01213.x

[B5] Persson E Berndtsson I Carlsson E Hallen AM Lindholm E Stoma-related complications and stoma size - a 2-year follow up Colorectal Dis 2010 12 971 976 19519689 10.1111/j.1463-1318.2009.01941.x

[B6] Parmar K Zammit M Smith A Kenyon D Lees N A prospective audit of early stoma complications in colorectal cancer treatment throughout the Greater Manchester and Cheshire colorectal cancer network Colorectal Dis 2011 13 935 938 20478001 10.1111/j.1463-1318.2010.02325.x

[B7] Nguyen MH Pittas F How large should a skin trephine be for an end stoma? Aust N Z J Surg 1999 69 675 676 10515344 10.1046/j.1440-1622.1999.01663.x

[B8] Keighley MR Williams NS Surgery of the Anus, Rectum and Colon 2008 Philadelphia, PA: Saunders Elsevier

[B9] Carne PW Robertson GM Frizelle FA Parastomal hernia Br J Surg 2003 90 784 793 12854101 10.1002/bjs.4220

[B10] Pilgrim CH McIntyre R Bailey M Prospective audit of parastomal hernia: prevalence and associated comorbidities Dis Colon Rectum 2010 53 71 76 20010354 10.1007/DCR.0b013e3181bdee8c

[B11] Sjodahl R Anderberg B Bolin T Parastomal hernia in relation to site of the abdominal stoma Br J Surg 1988 75 339 341 2965951 10.1002/bjs.1800750414

[B12] Lian L Wu XR He XS Zou YF Wu XJ Lan P Wang JP Extraperitoneal vs. intraperitoneal route for permanent colostomy: a meta-analysis of 1,071 patients Int J Colorectal Dis 2012 27 59 64 21892608 10.1007/s00384-011-1293-6

[B13] Serra-Aracil X Bombardo-Junca J Moreno-Matias J Darnell A Mora-Lopez L Alcantara-Moral M Ayguavives-Garnica I Navarro-Soto S Randomized, controlled, prospective trial of the use of a mesh to prevent parastomal hernia Ann Surg 2009 249 583 587 19300232 10.1097/SLA.0b013e31819ec809

[B14] Janes A Cengiz Y Israelsson LA Preventing parastomal hernia with a prosthetic mesh Arch Surg 2004 139 1356 1358 15613293 10.1001/archsurg.139.12.1356

[B15] Janes A Cengiz Y Israelsson LA Preventing parastomal hernia with a prosthetic mesh: a 5-year follow-up of a randomized study World J Surg 2009 33 118 121 discussion 122-113 19011935 10.1007/s00268-008-9785-4

[B16] Tam KW Wei PL Kuo LJ Wu CH Systematic review of the use of a mesh to prevent parastomal hernia World J Surg 2010 34 2723 2729 20661562 10.1007/s00268-010-0739-2

[B17] Shabbir J Chaudhary BN Dawson R A systematic review on the use of prophylactic mesh during primary stoma formation to prevent parastomal hernia formation Colorectal Dis 2012 14 931 936 21929523 10.1111/j.1463-1318.2011.02835.x

[B18] Janes A Cengiz Y Israelsson LA Experiences with a prophylactic mesh in 93 consecutive ostomies World J Surg 2010 34 1637 1640 20182719 10.1007/s00268-010-0492-6

[B19] Evans MD Williams GL Stephenson BM Preventing parastomal herniation: is prophylactic prosthetic mesh absolutely necessary? World J Surg 2009 33 1538 1539 author reply 1540-1531 19288283 10.1007/s00268-009-9972-y

[B20] Wang J Dou Z Wang T Liu D Wang L Circular stapler-assisted extraperitoneal colostomy Dig Surg 2010 27 521 524 21196736 10.1159/000321903

[B21] Resnick S New method of bowel stoma formation Am J Surg 1986 152 545 548 3777337 10.1016/0002-9610(86)90226-6

[B22] Wijeyekoon SP Gurusamy K El-Gendy K Chan CL Prevention of parastomal herniation with biologic/composite prosthetic mesh: a systematic review and meta-analysis of randomized controlled trials J Am Coll Surg 2010 211 637 645 20829077 10.1016/j.jamcollsurg.2010.06.111

[B23] Sajid MS Kalra L Hutson K Sains P Parastomal hernia as a consequence of colorectal cancer resections can prophylactically be controlled by mesh insertion at the time of primary surgery: a literature based systematic review of published trials Minerva Chir 2012 67 289 296 23022753

[B24] Helgstrand F Gogenur I Rosenberg J Prevention of parastomal hernia by the placement of a mesh at the primary operation Hernia 2008 12 577 582 18523836 10.1007/s10029-008-0387-8

[B25] Janes A Weisby L Israelsson LA Parastomal hernia: clinical and radiological definitions Hernia 2011 15 189 192 21188441 10.1007/s10029-010-0769-6

[B26] Gurmu A Gunnarsson U Strigard K Imaging of parastomal hernia using three-dimensional intrastomal ultrasonography Br J Surg 2011 98 1026 1029 21509751 10.1002/bjs.7505

[B27] Clavien PA Barkun J de Oliveira ML Vauthey JN Dindo D Schulick RD de Santibanes E Pekolj J Slankamenac K Bassi C Graf R Vonlanthen R Padbury R Cameron JL Makuuchi M The Clavien-Dindo classification of surgical complications: five-year experience Ann Surg 2009 250 187 196 19638912 10.1097/SLA.0b013e3181b13ca2

[B28] Angenete E Correa-Marinez A Heath J Gonzalez E Wedin A Prytz M Asplund D Haglind E Ostomy function after abdominoperineal resection - a clinical and patient evaluation Int J Colorectal Dis 2012 27 1267 1274 22451254 10.1007/s00384-012-1463-1

[B29] Bjoholt I Janson M Jonsson B Haglind E Principles for the design of the economic evaluation of COLOR II: an international clinical trial in surgery comparing laparoscopic and open surgery in rectal cancer Int J Technol Assess Health Care 2006 22 130 135 16673689 10.1017/s0266462306050926

[B30] Lopez-Cano M Lozoya-Trujillo R Quiroga S Sanchez JL Vallribera F Marti M Jimenez LM Armengol-Carrasco M Espin E Use of a prosthetic mesh to prevent parastomal hernia during laparoscopic abdominoperineal resection: a randomized controlled trial Hernia 2012 16 661 667 22782367 10.1007/s10029-012-0952-z

[B31] Correa Marinez A Bengtsson J Eriksson M Hjalmarsson Y Palmqvist E Sjögren P Jivegård L End colostomy - with or without mesh reinforcement [Terminal kolostomi - med eller utan nätförstärkning] 2013 Göteborg: Sahlgrenska Universitetssjukhuset HTA-centrum Regional activity-based HTA 2013:62. Västra Götalandsregionen

[B32] Peristomal Mesh for Prophylaxis of Parastomal Hernia http://clinicaltrials.gov/show/NCT00496418

[B33] Prevention of Parastomal Hernia by Primary Mesh Insertion http://clinicaltrials.gov/show/NCT00641342

[B34] Trial Concerning the Frequency of Parastomal Hernia With or Without a Mesh (STOMAMESH) http://clinicaltrials.gov/show/NCT00917995

[B35] Role of Prosthetic Mesh in Preventing Parastomal Hernias (RPMPPH) http://clinicaltrials.gov/ct2/show/NCT01955278

[B36] Prospective Study of the Use of a Mesh to Prevent Parastomal Hernia after Laparoscopic Abdominoperineal Resection http://clinicaltrials.gov/ct2/show/NCT01722565

[B37] Primary Prevention of Peristomial Hernias via Parietal Prostheses http://clinicaltrials.gov/ct2/show/NCT01380860 10.1016/j.dld.2016.03.020 27130912

[B38] Brandsma HT Hansson BM Haan HV Aufenacker TJ Rosman C Bleichrodt RP PREVENTion of a parastomal hernia with a prosthetic mesh in patients undergoing permanent end-colostomy; the PREVENT-trial: study protocol for a multicenter randomized controlled trial Trials 2012 13 226 23186083 10.1186/1745-6215-13-226 PMC3576295

